# Reduced Bone Quality of Sacrum and Lumbal Vertebrae Spongiosa in Toll-like Receptor 2- and Toll-like Receptor 4-Knockout Mice: A Blinded Micro-Computerized Analysis

**DOI:** 10.3390/biom15020239

**Published:** 2025-02-07

**Authors:** Kilian Roth, Johannes Dominikus Pallua, Gerald Degenhart, Tobias De Zordo, Christian Kremser, Christian Reif, Werner Streif, Michael Schirmer

**Affiliations:** 1 Department of Internal Medicine, Medical University of Innsbruck, 6020 Innsbruck, Austria; kilian-roth@gmx.de; 2 Core Facility of MicroCT, Clinic for Orthopedics and Traumatology, Medical University of Innsbruck, 6020 Innsbruck, Austria; johannes.pallua@i-med.ac.at; 3 Core Facility of MicroCT, University Clinic for Radiology, Anichstraße 35, 6020 Innsbruck, Austria; gerald.degenhart@i-med.ac.at; 4 Department of Radiology, Medical University of Innsbruck, 6020 Innsbruck, Austria; tobias@de-zordo.net (T.D.Z.); christian.kremser@i-med.ac.at (C.K.); 5 Radiology Department, Brixsana Private Clinic, 39042 Brixen-Bressanone, Italy; 6 Department of Pediatrics I, Medical University of Innsbruck, 6020 Innsbruck, Austria; christian.reif@tirol-kliniken.at (C.R.); werner.streif@i-med.ac.at (W.S.); 7 Office for Internal Medicine/Rheumatology, 6060 Hall, Austria

**Keywords:** toll-like receptors, micro-computed tomography, bone microarchitecture, spondyloarthritis, bone remodeling, osteoclastogenesis, infection

## Abstract

Toll-like receptors (TLRs) are pivotal in modulating immune responses and have been implicated in bone remodeling. This in vivo study investigates the impact of TLR2 and TLR4 signaling on trabecular bone structure using micro-computed tomography in a murine model. Sacrum and lumbar vertebrae (L5, L6) from wildtype (WT), TLR2-knockout (TLR2-KO), and TLR4-knockout (TLR4-KO) mice were analyzed, with trabecular parameters such as connectivity density (Conn-Dens), trabecular thickness (DT-TbTh), and variability metrics (DT-Tb,(1/N),SD and DT-TbThSD) assessed. The results revealed significant differences among genotypes: TLR4-KO mice exhibited increased variability in trabecular distribution, indicating less stable bone structures, while TLR-KO mice showed lower variability in trabecular thickness, suggesting enhanced uniformity and robustness. BV/TV and 3D reconstructions highlighted lower bone volume fractions in the sacrum compared to lumbar vertebrae across genotypes, consistent with human observations of reduced sacral bone volume in spondyloarthritis (SpA). Interestingly, bone changes were independent of immunization-induced SpA, emphasizing a direct role in TLR signaling. These findings provide novel insights into the role of TLRs in bone microarchitecture and suggest implications for bone-related pathologies, particularly those involving inflammatory pathways. Future research may explore the translational relevance of TLR-mediated mechanisms in osteopenia and osteoporosis.

## 1. Introduction

Reactive arthritis is considered a subtype of spondyloarthritis (SpA) with an infectious trigger, followed by inflammatory arthritis [1]. So far, Gram-negative bacteria such as *Salmonella*, *Shigella* or *Yersinia*, *Klebsiella*, *Chlamydiae*, *Mycobacterium hominis*, *Ureaplasma urealyticum* and even respiratory infections in children were discussed as possible infectious triggers [2,3,4,5,6]. Consequently, Zeidler and Hudson proposed a polymicrobial/co-infective approach, requiring a holistic data collection of urogenital, respiratory and enteric pathogens to answer this critical question [7]. For animal in vivo studies, an immunized mouse model was established to further examine different pathophysiological aspects of SpA [8].

Within the innate immune system, toll-like receptors (TLRs) play a key role and are usually expressed on macrophages and dendritic cells. Earlier studies, however, also showed that additional expression of TLRs on pro-inflammatory T-cells is an exciting concept for a potential link between infections and the adaptive immune system. TLRs recognize structurally conserved molecules from bacteria, viruses, fungi, and parasites. Despite the protective function of TLRs as part of the innate immune system, aberrant responses of TLRs contribute to inflammatory and autoimmune disorders [9]. Indeed, circulating pro-inflammatory CD28^null^ T-helper cells express both TLR2 and TLR4 in SpA disease [10]. Agonistic TLR-ligands include lipopolysaccharides (LPSs) derived from Gram-positive bacteria for binding to TLR2, and Gram-negative bacteria for binding to TLR4 [11]. After TLR ligation, effects vary between the ligation of TLRs, leading to impaired T-regulatory cells with immunosuppressive properties [12] and TLR4-primed human mesenchymal stem cells switching into a pro-inflammatory state [13]. From an osteological perspective, it is interesting to read that an LPS as a TLR-agonist induces osteoclast formation and bone resorption in vitro and in vivo [14,15]. In vivo, *P. aeruginosa* LPS caused significant bone destruction, which was entirely prevented in mice without TLR4 expression [16]. Only recently, dysregulation of osteoblasts and osteoclasts induced by abnormal expression of TLR4 was described as the primary molecular mechanism underlying the pathological processes of osteoporosis, and clinical data were reviewed, showing that TLR4 polymorphisms and aberrant TLR4 expression were associated with the severity of osteoporosis [17]. In TLR2-knocked out (TLR2-KO) mice, however, tibiae and femora showed an increased bone volume of metaphyseal trabeculae and elevated numbers of tartrate-resistant acid phosphatase (TRAP)-positive osteoclasts, while the number of multinucleated TRAP-positive osteoclasts was reduced [18]. For ankylosing spondylitis, the most severe form of SpA with typical bone formation, a recent meta-analysis did not suggest any association of toll-like receptor 4 gene polymorphisms with susceptibility to ankylosing spondylitis [19,20], although some polymorphisms of TLR4 are linked to ankylosing spondylitis [21]. The definite role of TLRs, however, can only be defined in knockout (KO) animal models.

Focusing on the bone structures of the sacrum and the lumbal vertebrae appears to be the most important osteological aspect of axial SpA. The microstructure of the spinal bone has not been investigated in TLR-KO mice so far. Different parameters can be applied for quantification of bone, which are assessed by micro-computerized tomography (micro-CT). Micro-CT is a powerful imaging technology used extensively in biomedical research to evaluate high-resolution specimens. Micro-CT can achieve resolutions in the submicron range using a cone-beam X-ray tube and a rotating sample holder, allowing for detailed 3D imaging of various materials and tissues [22,23,24]. Unlike 2D imaging approaches, micro-CT enables the direct evaluation of 3D structures, facilitating more comprehensive analyses of complex biological specimens [25]. This technological advancement allowed for morphometric analysis and quantification, which were difficult to achieve with other imaging techniques [26,27,28]. Micro-CT has broad applications across numerous medical and scientific disciplines [29]. As technology advances, micro-CT’s applications and impact across medical fields and beyond are expected to expand, fostering scientific and technological innovation [30,31,32,33,34,35,36,37].

Therefore, this work aimed to study the roles of TLR2- and TLR4-expression in a murine model. Micro-computerized tomography (micro-CT) data of the sacrum and lumbal vertebrae were assessed in wildtype (WT), TLR2-KO, and TLR4-KO mice to identify possible TLR-mediated effects on bone formation in vivo, both in controls and a murine SpA-model.

## 2. Materials and Methods

### 2.1. Animal Model

A modified proteoglycan-induced spondylitis mouse model was applied to male BALB/c mice [8]. The genotype was either WT (from Charles River; Schweinfurt, Germany), TLR2-KO or TLR4-KO (both from Oriental BioServices Inc., Kyoto, Japan), with 15 mice in each group. Of these, 9 BALBc mice per genotype group were immunized with 100 µg of proteoglycan protein in 100 µL of N,N-dimethyl-N,N-dioctadecylammonium (DDA) [38]. At the end of this study, mice were sacrificed, and sacrum and lumbal vertebrae L5 and L6 were fixed in 4% paraformaldehyde (Sigma-Aldrich Handels Gmbh, Vienna, Austria). One mouse died early during the study.

Positive approval for this animal study was obtained from the national authorities (GZ. BMWFW-66.011/0137-WF/V/3b/2016, approval date: 2017-02-28).

### 2.2. Micro-Computerized Tomography

Micro-CT experiments of the fixed sacrum and lumbar vertebrae preparations were performed using a vivaCT 40 system (Scanco Medical AG, Brüttisellen, Switzerland). The scanning parameters included 1000 projections, achieving a 10.5 µm isotropic resolution within a 21.5 mm field of view. The tube settings comprised a voltage of 70 kV, a current of 114 µA, and an integration time of 200 ms per projection. The resulting images had a matrix size of 2048 × 2048 voxels with a 16-bit grayscale depth. The length of the image stack varied according to the size of the collected samples.

**Figure 1:**
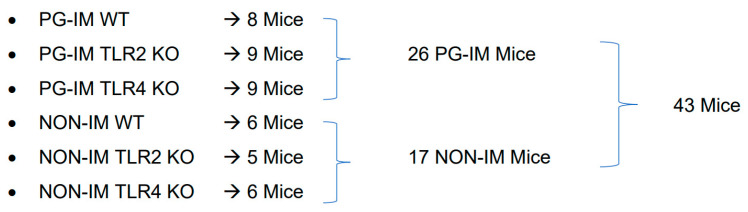
Summary of bone imaging groups analyzed by micro-computed tomography (micro-CT). A total of 43 mice were divided into two main categories of immunized (PG-IM) and non-immunized (NON-IM) mice: PG-IM (proteoglycan-induced inflammation), including wildtype (WT, 8 mice), TLR2-knockout (KO, 9 mice), and TLR4-knockout (KO, 9 mice) mice (26 mice in total), and NON-IM (non-inflamed controls), including wildtype (WT, 6 mice), TLR2-knockout (KO, 5 mice), and TLR4-knockout (KO, 6 mice) mice (17 mice in total).

The obtained dataset includes information on different genotypes (WT, TLR2-KO, TLR4-KO), their localization (1 = sacrum, 2 = L6, 3 = L5), and bone parameters such as BV/TV, Conn-Dens, SMI, Tb.N, Tb.Th, Tb.Sp, Tb.1/N.SD, Tb.Th.SD, and Tb.Sp.SD (abbreviations outlined in Table 1). For this experiment, the proteoglycan immunized group (PG-IM) and the control group (NON-IM) were each divided into three subgroups: wildtype (WT), toll-like receptor 2-knockout (TLR2-KO), and toll-like receptor 4-knockout (TLR4-KO). In total, µCT bone images were obtained for six groups, including PG-IM WT (8 mice), PG-IM TLR2-KO (9 mice), PG-IM TLR4-KO (9 mice), NON-IM WT (6 mice), NON-IM TLR2-KO (5 mice), and NON-IM TLR4-KO (6 mice), as summarized in Figure 1.

### 2.3. Image Reconstruction

Image reconstruction was performed using cone beam convoluted back-projection on the system workstation, which operates on the operating system openVMS^®®^ by HP (Hewlett Packard Enterprise, Houston, TX, USA) in conjunction with the Scanco Medical AG software suite µCT v6.1 (Scanco Medical AG, Brüttisellen, Switzerland). Post-processing involved two steps: the Image Processing Language (IPL) integrated alignment algorithm (Scanco Medical AG, Brüttisellen, Switzerland) was applied to longitudinally scanned samples to reorient them axially; and micro-CT images from the cancellous bone of the sacrum (localization 1) and lumbar vertebrae L6 (localization 2) and L5 (localization 3) were semi-automatically segmented from cortical bone. By following the guidelines for manual contouring and linear morphing by Scanco Medical, segmented bone areas were then analyzed using IPL for the bone parameters summarized in Table 1. The micro-CT images were blinded to the investigator (K.R.).

### 2.4. Statistical Analysis and 3D Reconstructions

Descriptive statistics, including the mean, median, and standard deviation, were computed for each combination of genotype and localization across all parameters. By examining each genotype–localization pairing, this analysis aimed to provide a foundational understanding of the data distribution for all parameters. A one-way ANOVA was performed to determine if there were significant differences between the bone parameters among the three genotypes (WT, TLR2-KO, and TLR4-KO) at each localization. Significant findings are reported with *p*-values, highlighting the differences between the parameters such as Conn-Dens, DT-Tb.N, DT-Tb.Th, DT-Tb.Sp,DT- Tb.Th.SD, and Mean2.

A two-way ANOVA was performed to assess the impact of immunization status (imm = 0, without immunization; imm = 1, with immunization) on bone structure parameters. This analysis considered both genotype and immunization status as factors and interaction effects were tested. Significant results were obtained for parameters like Conn-Dens, Tb.N, Tb.Th, Tb.Sp, Tb.1/N.SD, and Tb.Th.SD.

Following the ANOVA, Tukey’s Honest Significant Difference (HSD) test was used to perform pairwise comparisons among the genotypes. This post hoc analysis identified significant differences between genotype pairs (e.g., WT vs. TLR2-KO, WT vs. TLR4-KO, TLR2-KO vs. TLR4-KO).

Principal Component Analysis (PCA) was utilized to reduce the dimensionality of the data and identify the principal components that explain the most variance. Following PCA, KMeans clustering was applied to group the samples into distinct clusters based on their bone structure parameters. Clusters were characterized by their genotypic composition and immunization status, revealing natural groupings and underlying patterns in the data. All statistical analyses and visualizations were performed using GraphPad Prism software (version 10, San Diego, CA, USA) statistical software packages.

### 2.5. Visualizations

Boxplots and interaction plots were created to visually represent the significant differences and interactions identified in the statistical analyses. These plots highlighted the distribution of each parameter across genotypes and the effect of immunization status, illustrating significant differences and interactions.

## 3. Results

Micro-CT segments from the sacrum and the adjacent two lumbal vertebrae (L5 and L6) were analyzed from 15 WT, 14 TLR2-KO, and 15 TLR4-KO mice. Out of these, eight TLR2-KO mice and nine mice in each WT and the TLR4-KO groups were immunized according to the SpA protocol [8], and six mice remained non-immunized per group.

### 3.1. Reduced Quality of Bone Spongiosa in TLR-Knockout Compared to Wildtype Mice

**Figure 2:**
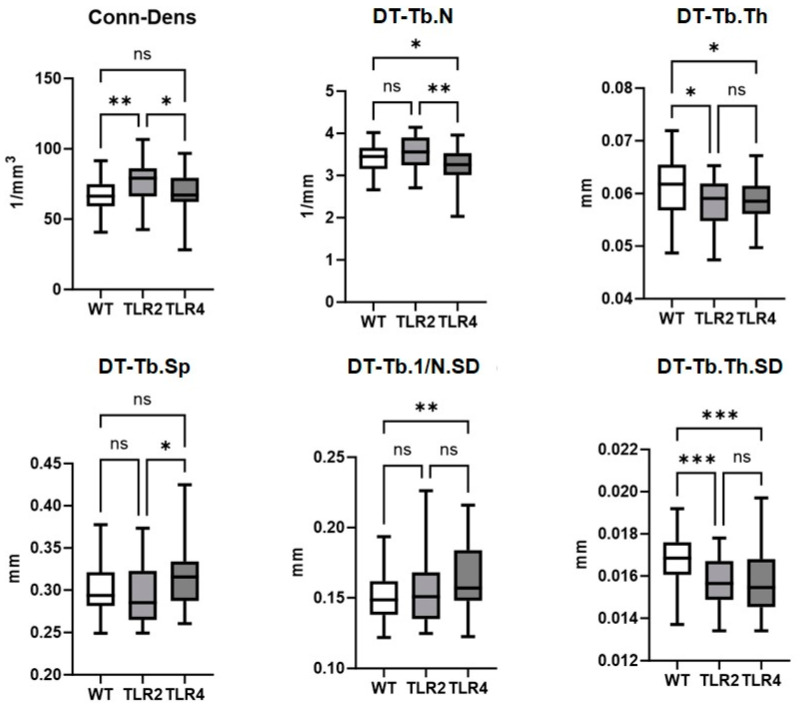
Boxplots illustrating the distribution of connectivity density (Conn-Dens) and trabecular bone parameters for WT, TLR2-KO, and TLR4-KO genotypes of micro-CT segments from the sacrum and the adjacent two lumbal vertebrae (L5 and L6). The symbol * (*p* < 0.05) indicates statistical significance, ** (*p* < 0.01) represents high significance, and *** (*p* < 0.001) signifies very high significance. ns suggests that the difference between the groups was insignificant (*p* > 0.05).

The results from one-way ANOVA indicate several differences in bone volume and structural parameters between the different genetic backgrounds. All trabecular parameters differ between WT and the TLR-KO mice (Figure 2 and Table 2). This first analysis of in vivo data describes less bone spongiosa with thinner trabeculae and less variability and uniformity of local thicknesses in TLR-KO than in WT mice. In addition, TLR4 mice have fewer trabeculae combined with a broader distance between the trabeculae in TLR-KO4 when compared to TLR2-KO mice.

#### 3.1.1. Connectivity Density

Connectivity density (Conn-Dens), the parameter for the degree of connectivity of trabeculae normalized by total volume is higher in TLR2-KO mice compared to both TLR4-KO (mean difference 7670/mm^3^) and WT mice (mean difference 9798/mm^3^). This shows that the connectivity of the trabecular network differs between TLR2-KO and TLR4-KO mice, indicating more mechanical strength in TLR2-KO mice than in TLR4-KO as in WT mice.

#### 3.1.2. Trabecular Number

The trabecular number (DT-Tb.N) is critical for bone strength and resilience. The number of trabeculae (DT-Tb.N) is lowest in TLR4-KO mice compared to TLR2-KO (mean difference −0.311/mm) and WT (mean difference −0.208/mm). This difference is significant between TLR2-KO vs. TLR4-KO (*p*: 0.001) and TLR4-KO vs. WT (*p*: 0.040).

#### 3.1.3. Trabecular Thickness

Concerning trabecular thickness (DT-Tb.Th), differences between TLR2-KO compared to WT (*p*: 0.021) and TLR4-KO compared to WT (*p*: 0.027) suggest that both TLR2 and TLR4 signaling have an impact on trabecular thickness. DT-TbTh is highest in WT mice compared to TLR2-KO (mean difference 0.003 mm) and TLR4-KO mice (mean difference 0.003 mm). This parameter is crucial as thinner trabeculae of the TLR-KO mice generally indicate weaker bones.

#### 3.1.4. Trabecular Separation

In line with the results listed above, trabecular separation (DT-Tb.Sp), the mean distance between the trabeculae, is increased in TLR4-KO compared to TLR2-KO (mean difference 0.031 mm) and WT mice (mean difference 0.026 mm). DT-Tb.Sp differs between TLR2-KO compared to TLR4-KO (*p*: 0.002) and TLR4 compared to WT (*p*: 0.013). The TLR4-KO status accompanies significantly reduced trabecular separation, resulting in a denser structure. This reduction in separation is beneficial for bone strength as it implies closer and more interconnected trabeculae.

#### 3.1.5. Standard Deviation of Trabecular Parameters

The significant differences observed among the genotypes in Figure 2 suggest that TLRs influence the variability and uniformity of trabecular bone structure. Specifically, the standard deviation of local inverse number (DT-Tb,1/N.SD) was higher in TLR4-KO mice than in the wildtype, indicating increased variability in trabecular distribution. This high variability is associated with less stable bone structures, as irregular trabecular spacing can compromise the mechanical integrity of the bone. Conversely, the lower variability in trabecular thickness (DT-Tb.Th.SD) observed in TLR-KO mice suggests a more uniform trabecular architecture. This consistency may enhance bone quality and mechanical robustness, as uniform trabecular elements distribute mechanical loads more evenly.

These findings underscore the intricate balance of TLR2 and TLR4 signaling in modulating the structural integrity of trabecular bone and its architectural consistency. The observed trends highlight the complexity of TLR-mediated pathways in bone remodeling and their potential implications for bone-related pathologies.

### 3.2. Decreased Bone Volume Fraction in the Sacrum Compared to Lumbal Vertebrae

**Figure 3:**
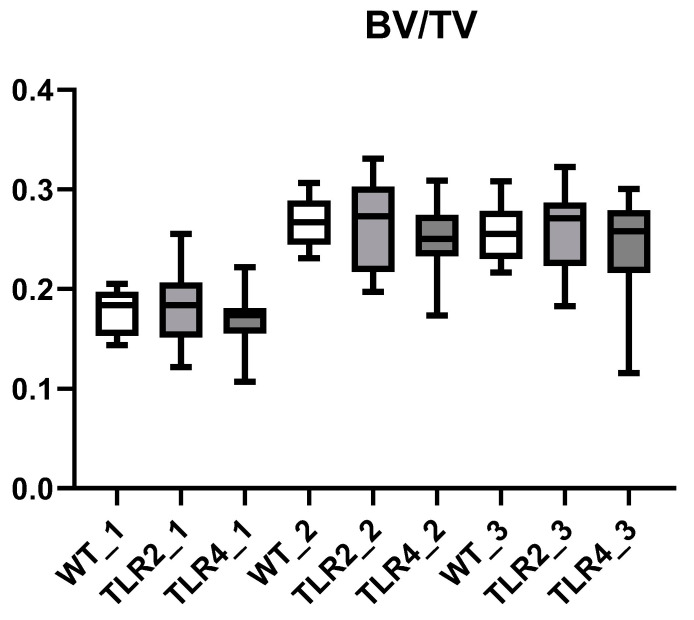
Comparison between bone volume fractions (BV/TV) in the sacrum and lumbal vertebrae (1, sacrum; 2, L6; 3, L5), for all genotypes (white, WT; black, TLR2-KO; gray, TLR4-KO). Differences between sacrum and lumbal vertebrae are all highly significant (*p* < 0.0001).

The bone volume fraction (BV/TV) as the ratio of segmented bone volume to total volume was lower in the sacrum than in the lumbal vertebrae L5 and L6 (*p*: <0.001), independent from the genotypes of WT, TLR2-KO, and TLR4-KO mice (Figure 3).

### 3.3. Effects of Prior Immunization on Bone Parameters in TLR2- and TLR4-Knockout Mice

**Figure 4:**
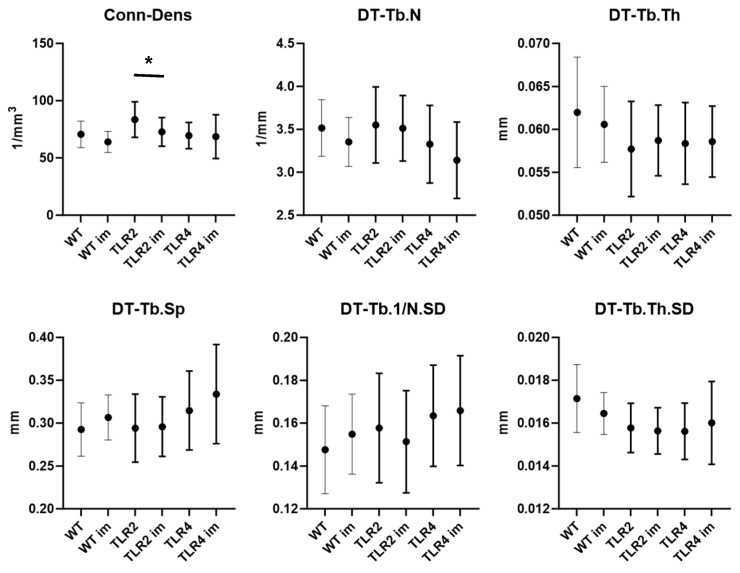
Interaction plots showing the effects of immunization status on key bone parameters depending on genetic background (WT, TLR2-KO, TLR4-KO). The only significant difference between non-immunized and immunized mice of the same WT or KO type was for Conn-Dens in TLR2-KO mice (*, with *p* = 0.0492 using Šídák’s multiple comparisons test).

Immunization significantly affects Conn-Dens with higher Conn-Dens in non-immunized TLR2-KO mice than in immunized (*p* = 0.0492) (Figure 4). All other findings were not significant.

### 3.4. Principal Component Analysis (PCA) and Clustering

PCA was utilized to reduce the dataset’s dimensionality and identify the primary components that explain the most variance among genotypes and bone structure parameters. The first two principal components (PCs) emerged as the key players, consistently accounting for the majority of the variance in the data. PC-1, with a consistent explanation of between 85.89% and 87.77%, and PC-2, contributing 9.53% to 11.56%, stood out across different genotype comparisons (WT vs. TLR2-KO, WT vs. TLR4-KO, and TLR2-KO vs. TLR4-KO) (Table 3). Key parameters such as connectivity density (Conn-Dens), Mean1, and Mean2 displayed high loadings, indicating their significant contribution to the observed variance.

PCA plots clearly differentiated the genotypes, highlighting differences in trabecular parameters such as Conn-Dens, trabecular number (DT-Tb.N), and trabecular thickness (DT-Tb.Th). The analysis also highlighted the significant variability in trabecular architecture, with TLR2-KO and TLR4-KO genotypes displaying more significant variability than WT mice. These findings align with the ANOVA results and support the hypothesis that TLR2 and TLR4 signaling pathways significantly influence the bone structure and quality.

**Figure 5:**
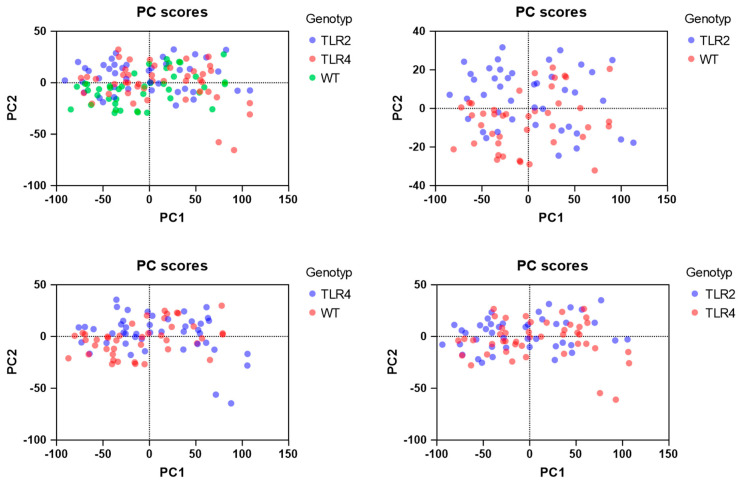
PCA boxplots showing the distribution of samples based on the first two principal components, color-coded by their genotype.

This PCA-based approach effectively highlights the intricate relationships between genetic background and bone microarchitecture, providing new insights into the role of TLR signaling in bone metabolism. The clustering analysis further underscores the distinct genotypic effects on bone density and structural uniformity. These findings contribute to a deeper understanding of how TLR pathways impact trabecular bone characteristics and inspire and motivate future investigations into their role in bone-related pathologies. The PCA results are illustrated in Figure 5.

### 3.5. Three-Dimensional Reconstructions of Bone from Different Locations and Genetic Background

**Figure 6:**
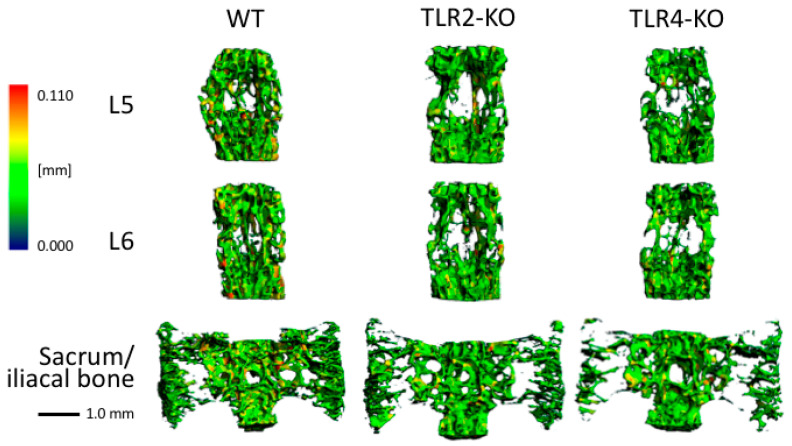
Three-dimensional reconstructions of µCT images from mice with the lowest BV/TV per group for different localizations and genetic backgrounds. Colors represent the Hounsfield scale applied for trabecular thickness (from blue = 0.000 mm to red = 0.110 mm). The line indicates the unit of length in mm.

The 3D reconstructions of trabecular bone depicted in Figure 6 unveiled significant differences across genotypes, particularly in the sacrum and lumbar vertebrae (which confirmed data from Figure 3). These reconstructions have shown that the sacrum generally exhibits lower bone volume fractions (BV/TV) compared to lumbar vertebrae (L5 and L6), a consistent pattern across WT, TLR2-KO, and TLR4-KO mice. Furthermore, the significantly greater trabecular thickness (DT-TbTh) in WT mice than in TLR2-KO and TLR4-KO mice underscores the robustness of the trabecular structure in WT mice, a finding of great significance in bone biology and genetics.

## 4. Discussion

This is the first in vivo study on µCT bone density measurements comparing WT with TLR2-KO and TLR4-KO BALB/c mice. It clearly shows the significant role of TLR2 and TLR4-signaling in bone metabolism. In detail, the number of trabeculae in TLR4-KO mice is lower than in WT and TLR2-KO mice; in line with this finding the separation of trabeculae is more extensive in TLR4-KO than in TLR2-KO mice (Figure 2). Also, the trabecular thickness in TLR4-KO mice is less than in WT mice, implying an osteopenic pattern in TLR-KO mice without TLR4-stimuli. In the literature, it is still undecided whether TLRs increase or inhibit osteoclast formation and bone resorption [14]. The role of TLRs is usually seen in the context of TLR-mediated osteoclast differentiation and bone resorption in inflammatory diseases, such as periodontal diseases [41]. Also, in humans, both TLR4 polymorphisms and aberrant expression of TLR4 have been associated with the clinical significance of osteoporosis in clinical practice [17]. In line with this, TLRs have also been shown on pro-inflammatory senescent T-helper cells from SpA patients [8], and they play a role in the cytokine network involved in SpA disease [42,43]. In vitro, TLR4-priming with bacterial lipopolysaccharides in MSCs leads to pro-inflammatory properties [13]. This in vivo KO model further suggests a specific role of TLR-mediated stimuli for increasing trabecular thickness, independent from the localization in the sacrum or the vertebral bodies, and with bone volume fractions (BV/TV) significantly lower in the sacrum than in the lumbar vertebral bodies. Similar but less significant data were found for TLR2-KO mice, which appear to be less dependent on TLR-mediated stimuli than the TLR4-KO mice. Also, the connectivity of trabecular structure was highest in TLR2-KO mice compared to both TLR4-KO and WT mice, suggesting a compensatory mechanism involved in murine bones without TLR-signaling.

The significant differences in standard deviations suggest that TLR signaling also plays a crucial role in maintaining the stability of the trabecular bone structure. For example, the high variability observed in DT-Tb,(1/N), SD for TLR4-KO compared to WT mice indicates less stable bone structures, as irregular trabecular spacing can reduce mechanical integrity. Conversely, the lower variability in DT-TbThSD for both TLR2-KO and TLR4-KO mice suggests a more consistent and uniform trabecular architecture, which suggests the complete restructuring of bone to the lower and weaker level in TLR-KO mice. These findings underscore the profound impact of TLR-mediated pathways on the microstructural characteristics and mechanical quality of bone. The 3D reconstructions also visually support the statistical results, showing a more uniform and interconnected trabecular network in WT mice compared to the disrupted and less dense structures in TLR2-KO and TLR4-KO mice.

Another clinically significant finding was that bone volume expressed as BV/TV was lower in the sacrum than in the lumbar vertebrae L6 and L5 (Figure 2 and Figure 5). These differences may occur because of different levels of mechanical stress or needs for vascular nutrition. Interestingly, this finding of reduced bone volume in the sacrum may further explain why, in patients with SpA, bone marrow edema is more frequently observed in the sacrum than in the vertebral bodies [44]. The typical Romanus lesions as signs of axial SpA-disease present as vertebral bone marrow edema only at the edges of the vertebral endplates, derived from the epiphyseal ring in juvenile patients [45].

All the effects mentioned above in TLR2-KO and TLR4-KO genotypes were independent of prior SpA-immunization, with key structural parameters showing no significant differences between immunized and non-immunized groups (Figure 3). This indicates that SpA-immunization does not affect these changes. An activating effect of immunization was described in the literature on bone marrow eosinophils for plasma cell survival [46], and periodontal bone loss was described only in rats after immunization with Actinomyces viscosus [47]. It implies that the immune system’s activation can directly or indirectly influence bone microarchitecture, but its impact might be more localized or specific to particular structural features. This could be due to inflammatory cytokines locally released during an immune response, enhancing our understanding of SpA and its relationship with immunization [28].

The most important limitation of this work is the missing comparison with TLR-KO mice other than TLR2-KO and TLR4-KO mice and the lack of studies on the exposure of WT mice to well-defined bacterial triggers as a positive control. However, such an approach leads to many other immunological reactions which cannot be foreseen. The specific effects of such bacterial triggers on osteoblasts and osteoclasts have already been described in vitro, and these would undoubtedly result in difficulties when interpreting bone data. Also, BALB/c mice are considered Th2-prone, and data may vary in Th1-prone mice. Further immunohistochemical and 2D histomorphometric techniques will be applied in future studies. Because of the methodological approach of micro-CTs, this study did not investigate the compact bone for direct comparison with the trabecular bone.

## 5. Conclusions

This in vivo study demonstrates that TLR2 and TLR4 signaling influences the trabecular bone structure, affecting both microstructural characteristics and the mechanical quality of bone. TLR-KO mice showed an osteopenic pattern, especially without TLR4-stimuli. Concerning variability and uniformity, TLR4-KO mice displayed increased variability in trabecular parameters, indicative of less stable bone architecture, while TLR2-KO mice exhibited reduced variability in trabecular thickness, correlating with more robust bone quality. Notably, bone volume fractions were consistently lower in the sacrum than in lumbar vertebrae, independent of immunization-induced SpA.

These findings underscore the pivotal role of TLR pathways in bone remodeling and lay the groundwork for understanding the direct involvement of infectious diseases on pathologic bone changes. The observed differences across genotypes underscore the potential of targeting TLR signaling as a promising therapeutic avenue for conditions such as osteopenia and osteoporosis. Further investigations are warranted to unravel the mechanisms underlying TLR-mediated effects and their translational relevance to human bone pathologies, offering hope for more effective treatments in the future.

## Figures and Tables

**Table 1:** Abbreviations, descriptions, and standard units for key micro-CT parameters—data adapted from [39,40]. BV/TV, DT-Tb.N, DT-Tb.Th and DT-Tb.Sp are recommended for the assessment of trabecular bone.

Measures	Description	Standard Unit
Bone volume ratio	Ratio of bone volume to total volume in ROI	%
Connectivity density	Interconnectivity of bone, normed by TV	1/mm^3^
Structure model index	0 for parallel plates, 3 for cylindrical rods	0 to 3
Trabecular number	Mean number of trabeculae per unit length	mm
Trabecular thickness	Mean thickness of trabeculae	mm
	SD of local thicknesses	mm
Trabecular separation	Mean distance between trabeculae	mm
	SD of local separations	mm
Volume of interest	Bone and background depending on calibration (e.g., for HA bone mineral density)	[mg HA/ccm], Lin.att. coefficient [1/cm]
		Hounsfield unit [HU], native file number [1]
Segmented region	Lin.att. of what was considered bone	Lin.att. coefficient [1/cm]

HA, hydroxyapatite; HU, Hounsfield unit; Lin.att., linear attenuation; ROI, region of interest; SD, standard deviation; TV, total volume. BV/TV, bone volume ratio; DT-Tb.N, trabecular number; DT-Tb.Th, trabecular thickness; DT-Tb.Sp, trabecular separation.

**Table 2:** Micro-CT parameters to characterize bone tissue. These parameters have relevant applications within medical diagnostics. Their diagnostic importance is determined using an ordinary one-way ANOVA, where * (*p* < 0.05) indicates statistical significance, ** (*p* < 0.01) reflects high significance, and *** (*p* < 0.001) signifies very high significance. ns demonstrates that the difference between the groups was insignificant (*p* > 0.05).

Name	Description	Unit	*p*-Values Based on an Ordinary One-Way ANOVA		
			WT vs. TLR2	WT vs. TLR4	TLR2 vs. TLR4
Conn-Dens	connectivity density, normed by TV	1/mm^3^	0.0051 **	0.7618 ^ns^	0.0330 *
DT-Tb.N	trabecular number	1/mm	0.4616 ^ns^	0.0398 *	0.0010 *
DT-Tb.Th	trabecular thickness	mm	0.0210 **	0.0265 *	0.9903 ^ns^
DT-Tb.Sp	trabecular separation = marrow thickness	mm	0.7447 ^ns^	0.1158 ^ns^	0.0195 *
DT-Tb.1/N.SD	standard deviation of local inverse number	mm	0.7520 ^ns^	0.0073 **	0.0520 ^ns^
DT-Tb.Th.SD	standard deviation of local thicknesses	mm	0.0007 ***	0.0008 ***	0.9953 ^ns^

TV, bone volume ratio; Conn-Dens, connectivity density; DT-Tb.N, trabecular number; DT-Tb.Th, trabecular thickness; DT-Tb.Th.SD, standard deviation of trabecular thickness; DT-Tb.Sp, trabecular separation.

**Table 3:** PCA of 3 genotypes. Figure 4 displays the PCA plots.

Genotype	PCA
**WT vs. TLR2 vs. TLR4**	PC-1 (86.39%) PC-2 (10.72%)
**WT vs. TLR2**	PC-1 (87.77%) PC-2 (9.53%)
**WT vs. TLR4**	PC-1 (85.89%) PC-2 (11.56%)
**TLR2 vs. TLR4**	PC-1 (86.75%) PC-2 (9.94%)

## Data Availability

The raw data supporting the conclusions of this article will be made available by the authors on reasonable request.
